# Supplementary material for: Predicting sepsis-related mortality and ICU admissions from telephone triage information of patients presenting to out-of-hours GP cooperatives with acute infections: A cohort study of linked routine care databases
Source: PLoS One. 2023 Dec 13;18(12):e0294557. doi: 10.1371/journal.pone.0294557 (PMC10718413; doi:10.1371/journal.pone.0294557)
Supplement: S1 Appendix — (DOCX) [file pone.0294557.s001.docx]

**S1 Appendix. Urgency and entry complaints**

The triage system that is used in Dutch out-of-hours GP cooperatives is the Netherlands Triage Standard. Triagists begin with a mandatory 'ABCD' check (Airway, Breathing, Circulation, Disability). This check determines life-threatening problems for which an ambulance must be sent directly. In case of no direct life-threatening situation, the triagist chooses an entry complaint to continue the urgency evaluation. Each entry complaint contains hierarchically ordered triage questions. Based on the triage nurses' responses, the NTS automatically generates an urgency level ranging from U0 to U5. This urgency level tells the triagist the response time within which a patient should receive a medical assessment (see Table A1). The triagist can overrule the automatically generated urgency allocation.

**Table A1. Description of urgency levels of the Netherlands Triage Standard**

| **Urgency Level** | **Title** | **Description** | **Response time** |
| --- | --- | --- | --- |
| U0 | Resuscitation | Failure of vital functions | Immediate |
| U1 | Life-threatening | Vital functions are unstable | As soon as possible |
| U2 | Emergent | Vital functions are threatened | Within 1 hour |
| U3 | Urgent | Risk for damage | Within a few hours |
| U4 | Non-urgent | Negligible risk for damage | Within 24 hours |
| U5 | Advice | No risk for damage | Next working day |

Table A2 gives an overview of included and excluded entry complaints in the study. For the excluded entry complaints, the risk of a serious course of an infection was assumed to be very low.

**Table A2. Included and excluded entry complaints**

| **Included** | **Excluded** |
| --- | --- |
| ABCD unstable | Abdominal pain child |
| Abdominal pain adult | Allergic reaction or insect bite |
| Arm or leg complaints | Burn |
| Back pain | Child-birth |
| Collapse or fainting | Death |
| Cough | Drowning |
| Diabetes | Ear complaints |
| Diarrhoea | Eye complaints |
| Dizziness | Fever child |
| Drain or probe | Foreign body |
| Fever adult | Ill child |
| General malaise | Implantable cardioverter-defibrillator |
| Genital complaints | Intoxication |
| Headache | Nose bleeding |
| Inflammation of skin or breast | Pregnancy |
| Seizure | Resuscitation |
| Neck complaints | Self-care advice |
| Neurological deficit | Teeth complaints |
| Obstipation | Trauma abdomen |
| Palpitations | Trauma back |
| Rectal complaints | Trauma extremity |
| Shortness of breath | Trauma face |
| Strange or suicidal behaviour | Trauma head |
| Thorax pain | Trauma neck |
| Throat complaints | Trauma thorax |
| Urinary problems | Trauma unspecified |
| Vomiting | Vaginal bleeding |
|  | Wound |
