# Supplementary material for: Predicting sepsis-related mortality and ICU admissions from telephone triage information of patients presenting to out-of-hours GP cooperatives with acute infections: A cohort study of linked routine care databases
Source: PLoS One. 2023 Dec 13;18(12):e0294557. doi: 10.1371/journal.pone.0294557 (PMC10718413; doi:10.1371/journal.pone.0294557)
Supplement: S2 Appendix — (DOCX) [file pone.0294557.s002.docx]

**S2 Appendix. Criteria for terminal patients**

Terminal patients were excluded because a terminal illness is a reason not to admit a patient to the hospital/ICU. A patient was considered terminal when:

1) The index contact was a home visit, and the patient died within three months after the home visit, without a hospital admission in this period.

AND

2) One or more of the following situations:

a) Dormicum (ATC N05CD08) parenterally was prescribed by own GP

OR

b) Known with malignancy in own general practice OR ICPC* code of index contact concerns malignancy AND patient has had contact with the own GP because of malignancy within 14 days before index contact

OR

c) ICPC code of index contact is A20 - conversation about euthanasia

OR

d) ICPC code of index contact is A96 - natural death

*ICPC codes related to malignancy:

A79, B72-B74, D74-D77, F74, H75, K72, L71, N74, R84, R85, S77, T71, U75-U77, U79, W72, X75-X77, Y77, Y78.
