# Supplementary material for: Predicting sepsis-related mortality and ICU admissions from telephone triage information of patients presenting to out-of-hours GP cooperatives with acute infections: A cohort study of linked routine care databases
Source: PLoS One. 2023 Dec 13;18(12):e0294557. doi: 10.1371/journal.pone.0294557 (PMC10718413; doi:10.1371/journal.pone.0294557)
Supplement: S3 Appendix — (DOCX) [file pone.0294557.s003.docx]

**S3 Appendix. Criteria for infectious condition**

We used a composite primary outcome, "sepsis-related adverse outcome", defined as:

1) hospital admission with at least one day at ICU of a patient with an infectious condition; admission is within 72 hours after contact with OOH GP cooperative

OR

2) death of a patient with an infectious condition; death is within 30 days after contact with OOH GP cooperative

Ad 1.

Patient has an infectious condition when the discharge diagnosis or primary diagnosis concerns an infection (ICD-10: International Classification of Diseases and Related Health Problems)*

Ad 2.

For patients without a hospital admission: a patient has an infectious condition when the ICPC code** of the OOH GP cooperative contact concerns an infection or the cause of death involves an infection (ICD-10***).

For patients with hospital admission: the patient has an infectious condition when the discharge diagnosis or primary diagnosis concerns an infection (ICD-10*)

* ICD-10 codes of hospital discharge diagnosis or main hospital diagnosis related to infection:

A04.5, A04.7, A08.3, A08.4, A09.0, A09.9, A31.0, A39.0, A39.2, A39.8, A40.0, A40.1, A40.3, A40.8, A40.9, A41.0, A41.5, A41.8, A41.9, A46, A48.1, A48.3, A49.0, A49.1, A49.2, A49.8, A49.9, A87, B01.2, B02.8, B02.9, B24, B25.9, B34.9, B37.1, B44.1, B99, G00.1, G00.9, G03.8, G03.9, H65.1, I33, I38, J04.0, J05.1, J06.9, J09, J10.0, J10.1, J10.8, J11.1, J12.3, J12.8, J12.9, J13, J14, J15.1, J15.2, J15.4, J15.5, J15.8, J15.9, J16.8, J18.1, J18.8, J18.9, J20.9, J21.9, J22, J32.9, J39.0, J44.01-J44.04, J44.09, J85.2, K10.2, K12.2, K25.1, K25.5, K26, K35.3, K35.8, K52.1, K57.2, K57.4, K57.8, K61.0, K63.1, K65.0, K65.9, K80.0, K80.1, K80.3, K80.4, K81, K81.8, K81.9, K83.0, L02.1, L02.3, L03.1, L73.2, O23.4, M46.36, M60.05, M72.63, M72.65, M72.68, M86.67, N39.0, N41.9, N45.9, N49.8, O85, R50.9, T81.4, T82.6, T82.7, T84.5. This list is based on the ICD-10 codes of the patients in our data set. It is not a complete list of all ICD-10 codes related to infection.

**ICPC codes of the OOH GP cooperative contact related to infection:

A02, A03, A71-A78, A92, B70, B90, D22, D70-D73, D88, D92, D98, D99, H70-H74, K70, L70, N70-N73, R70-R78, R80-R83, S09, S10, S11, S76, U70, U71, W70, W71, W94, X70, X71, X74, Y70-Y75. This list is based on the ICPC codes of the patients in our data set. It is not a complete list of all ICPC codes related to infection.

***ICD-10 codes of causes of death related to infection:

A021, A099, A169, A178, A409, A410, A415, A419, A46, A483, A490, A499, B004, B010, B029, B201, B348, B91, B99, G001, G039, H669, I339, I38, J100, J101, J108, J110, J111, J152, J182, J189, J209, J22, J440, J869, K255, K265, K578, K631, K658, K659, K803, K810, K819, K822, K830, L021, L022, L031, L039, L089, N10, N300, N390, N492. This list is based on the ICD-10 codes of the patients in our data set. It is not a complete list of all ICD-10 codes related to infection.
