# Supplementary material for: Predicting sepsis-related mortality and ICU admissions from telephone triage information of patients presenting to out-of-hours GP cooperatives with acute infections: A cohort study of linked routine care databases
Source: PLoS One. 2023 Dec 13;18(12):e0294557. doi: 10.1371/journal.pone.0294557 (PMC10718413; doi:10.1371/journal.pone.0294557)
Supplement: S4 Appendix — (DOCX) [file pone.0294557.s004.docx]

**S4 Appendix. Details of machine learning models**

For development of the gradient boosted random forest the hyperparameter settings were:

Maximum tree depth (tuned): {2, 3, 4, 5, 6}

Gamma (tuned): {0.0005, 0.001, 0.005, 0.01, 0.1}

Percentage of predictors sampled for development of each tree: {20%, 30%, 40%, 50%, 60%}

Percentage of data sampled to develop each tree: 90%

Number of trees: 250

Number of boosting rounds: 10

Loss function: cross entropy

For development of the feed-forward neural network the hyperparameter settings were:

Loss function: cross entropy

Number of hidden layers (tuned): {1, 2, 3}

Learning rate (tuned): {0.001, 0.0001}

Dropout regularisation rate (tuned): {0.3, 0.4, 0.5}

Batch size (tuned): {256, 512}

Optimiser: Adam (Kingma DP, Ba J. arXiv preprint arXiv:1412.6980. 2014).

Stopping criterium: early stopping (patience of 10 epochs).

Batch normalizsation was used. The hidden layer size was sequentially decreased by half across the network. The dropout was added to the input of each successive layer.
