# Supplementary material for: Predicting sepsis-related mortality and ICU admissions from telephone triage information of patients presenting to out-of-hours GP cooperatives with acute infections: A cohort study of linked routine care databases
Source: PLoS One. 2023 Dec 13;18(12):e0294557. doi: 10.1371/journal.pone.0294557 (PMC10718413; doi:10.1371/journal.pone.0294557)
Supplement: S1 Fig — (DOCX) [file pone.0294557.s005.docx]

**S7 Figure. Importance matrix of the variables in the random forest (XGBoost) model.**

|  | 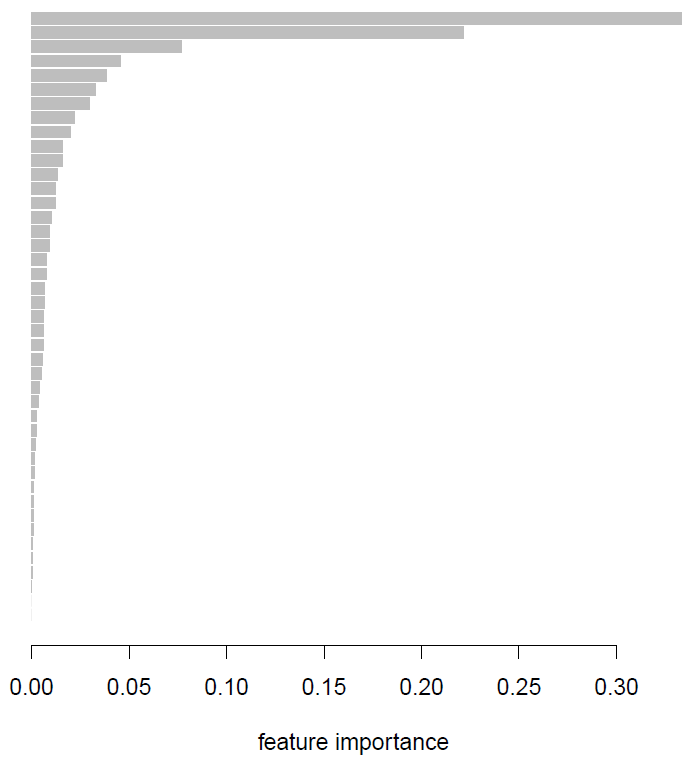 |
| --- | --- |
| Type of contact |  |
| Age |  |
| Number of comorbidities |  |
| Shortness of breath |  |
| General malaise |  |
| Urgency at triage |  |
| Previous contact with own GP |  |
| History of CVD |  |
| Immunosuppressive medication |  |
| Fever |  |
| History of COPD |  |
| History of Kidney disease |  |
| Sex |  |
| Time of contact |  |
| Thorax pain |  |
| History of diabetes |  |
| Number of entry complaints |  |
| Antibiotics prescribed <72h |  |
| ABCD unstable |  |
| Palpitations |  |
| Arm or leg complaints |  |
| Dizziness |  |
| Strange or suicidal behaviour |  |
| Abdominal pain adult |  |
| History of Malignancy |  |
| History of neurological disease |  |
| Headache |  |
| Back pain |  |
| Vomiting |  |
| Urinary problems |  |
| Cough |  |
| Neurological deficit |  |
| Inflammation of skin or breast |  |
| Rectal complaints |  |
| Diabetes |  |
| Previous contact with GP cooperative |  |
| Diarrhoea |  |
| Throat complaints |  |
| Obstipation |  |
| Collapse or fainting |  |
| Genital complaints |  |
| Seizure |  |
| Neck complaints |  |
|  |  |
|  |  |
|  |  |
|  |  |
