# Supplementary material for: Predicting sepsis-related mortality and ICU admissions from telephone triage information of patients presenting to out-of-hours GP cooperatives with acute infections: A cohort study of linked routine care databases
Source: PLoS One. 2023 Dec 13;18(12):e0294557. doi: 10.1371/journal.pone.0294557 (PMC10718413; doi:10.1371/journal.pone.0294557)
Supplement: S2 Fig — (DOCX) [file pone.0294557.s006.docx]

| 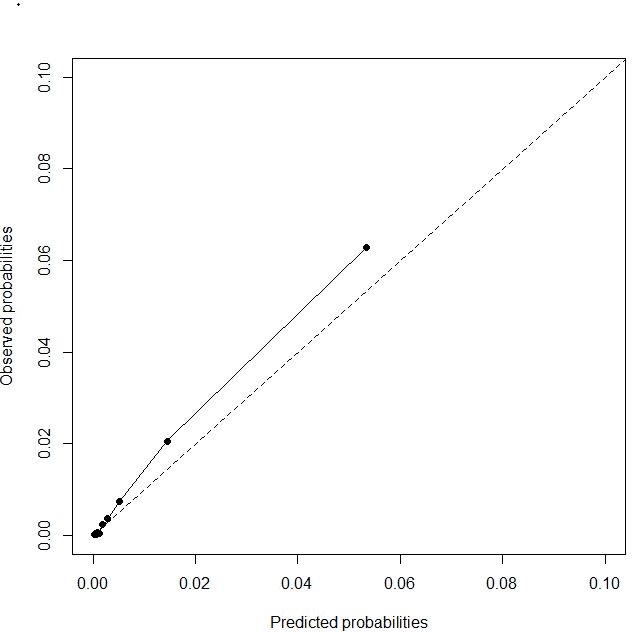 | 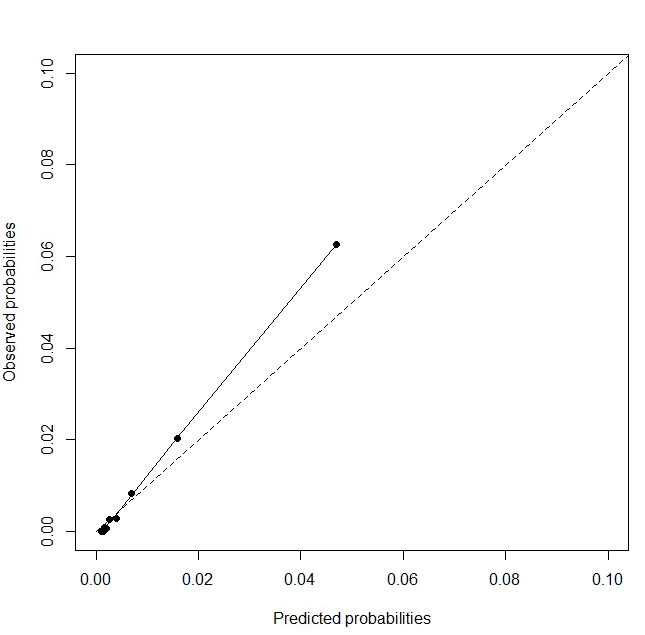 |
| --- | --- |
| A. Logistic regression model | B. Random forest ( XGBoost) model |
| 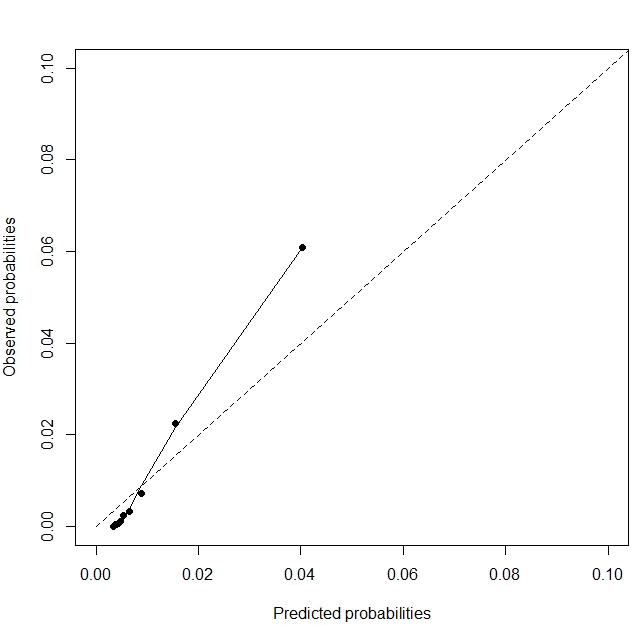 |  |
| C. Neural networks model |  |

**S8 Figure. Calibration plots of the developed models in the test data (n=50,932).**
