# Supplementary material for: Predicting sepsis-related mortality and ICU admissions from telephone triage information of patients presenting to out-of-hours GP cooperatives with acute infections: A cohort study of linked routine care databases
Source: PLoS One. 2023 Dec 13;18(12):e0294557. doi: 10.1371/journal.pone.0294557 (PMC10718413; doi:10.1371/journal.pone.0294557)
Supplement: S1 Table — (DOCX) [file pone.0294557.s007.docx]

**S5 Table. Relative risk (RR) of sepsis-related adverse outcomes for the 27 included entry complaints.**

| **Entry complaint** | **RR (95% CI)** |
| --- | --- |
| ABCD unstable | 7.65 (5.67 - 10.34) |
| General malaise | 4.00 (3.53 - 4.53) |
| Shortness of breath | 3.01 (2.78 - 3.26) |
| Diabetes | 2.61 (1.84 - 3.72) |
| Fever adult | 2.39 (2.01 - 2.84) |
| Drain or probe | 2.30 (1.03 - 5.15) |
| Strange or suicidal behaviour | 2.28 (1.82 - 2.86) |
| Vomiting | 1.63 (1.26 - 2.11) |
| Diarrhoea | 1.41 (0.94 - 2.09) |
| Neurological deficit | 1.07 (0.76 - 1.52) |
| Collapse or fainting | 0.96 (0.59 - 1.56) |
| Cough | 0.85 (0.65 - 1.10) |
| Obstipation | 0.75 (0.36 - 1.57) |
| Urinary problems | 0.67 (0.55 - 0.81) |
| Abdominal pain adult | 0.59 (0.51 - 0.69) |
| Seizure | 0.47 (0.07 - 3.37) |
| Neck complaints | 0.43 (0.16 - 1.15) |
| Arm or leg complaints | 0.43 (0.34 - 0.56) |
| Dizziness | 0.40 (0.26 - 0.62) |
| Back pain | 0.39 (0.26 - 0.59) |
| Thorax pain | 0.37 (0.28 - 0.49) |
| Rectal complaints | 0.28 (0.11 - 0.75) |
| Throat complaints | 0.25 (0.14 - 0.44) |
| Inflammation of skin or breast | 0.22 (0.15 - 0.34) |
| Headache | 0.18 (0.10 - 0.34) |
| Palpitations | 0.18 (0.09 - 0.34) |
| Genital complaints | 0.11 (0.03 – 0.43) |
