# Supplementary material for: Predicting sepsis-related mortality and ICU admissions from telephone triage information of patients presenting to out-of-hours GP cooperatives with acute infections: A cohort study of linked routine care databases
Source: PLoS One. 2023 Dec 13;18(12):e0294557. doi: 10.1371/journal.pone.0294557 (PMC10718413; doi:10.1371/journal.pone.0294557)
Supplement: S2 Table — (DOCX) [file pone.0294557.s008.docx]

**S6 Table. Crude and adjusted odds ratio (OR) of all variables included in the multivariable logistic regression model.**

| **Patient characteristic** | | **Crude OR (95%CI)** | **Adjusted OR (95%CI)** | **P-value** |
| --- | --- | --- | --- | --- |
| Male sex | | 1.54 (1.35 -1.79) | 1.35 (1.16 - 1.54) | <0.001 |
| Age | | 1.07 (1.06 - 1.07) | 1.03 (1.03 - 1.04) | <0.001 |
| Home visit | | 17.78 (14.83 - 21.33) | 4.38 (3.51 - 5.47) | <0.001 |
| Urgency at triage | | 0.44 (0.4 - 0.49) | 0.69 (0.61 - 0.79) | <0.001 |
| Moment of contact (ref.=day) | |  |  | 0.075 |
|  | evening | 1.13 (0.96 - 1.32) | 1.2 (1.02 - 1.42) | 0.03 |
|  | night | 1.33 (1.09 - 1.63) | 1.19 (0.97 - 1.47) | 0.094 |
| Number of entry complaints | | 0.98 (0.84 - 1.14) | 0.78 (0.65 - 0.95) | 0.011 |
| Previous GP contact | | 2.81 (2.44 - 3.23) | 1.6 (1.37 - 1.87) | <0.001 |
| History of COPD | | 3.8 (3.26 - 4.44) | 1.14 (0.96 - 1.35) | 0.14 |
| History of diabetes | | 3.04 (2.63 - 3.51) | 1.16 (0.99 - 1.36) | 0.06 |
| History of neurological disease | | 2.59 (2.06 - 3.26) | 1.48 (1.17 - 1.88) | 0.001 |
| History of kidney disease | | 4.1 (3.47 - 4.86) | 1.29 (1.07 - 1.55) | 0.006 |
| Immunosuppressive medication | | 3.82 (3.24 - 4.51) | 1.4 (1.17 - 1.68) | <0.001 |
| General malaise | | 4.7 (3.9 - 5.66) | 2.82 (2.25 - 3.52) | <0.001 |
| Vomiting | | 1.62 (1.14 - 2.28) | 1.45 (0.99 - 2.11) | 0.055 |
| Diabetes | | 3.13 (2.04 - 4.81) | 1.68 (1.05 - 2.67) | 0.029 |
| Diarrhoea | | 1.67 (1.04 - 2.67) | 1.64 (0.99 - 2.7) | 0.055 |
| Dizziness | | 0.41 (0.24 - 0.71) | 0.44 (0.25 - 0.78) | 0.005 |
| Genital complaints | | 0.09 (0.01 - 0.62) | 0.31 (0.04 - 2.19) | 0.237 |
| Palpitations | | 0.15 (0.06 - 0.37) | 0.24 (0.1 - 0.59) | 0.002 |
| Headache | | 0.2 (0.09 - 0.41) | 0.42 (0.2 - 0.91) | 0.027 |
| Throat complaints | | 0.28 (0.15 - 0.54) | 1.77 (0.89 - 3.5) | 0.103 |
| Fever | | 2.67 (2.11 - 3.39) | 2.24 (1.7 - 2.94) | <0.001 |
| Shortness of breath | | 4.03 (3.49 - 4.66) | 1.81 (1.48 - 2.2) | <0.001 |
| Neurological deficit | | 1.02 (0.64 - 1.63) | 0.63 (0.39 - 1.04) | 0.069 |
| Thorax pain | | 0.37 (0.26 - 0.52) | 0.53 (0.36 - 0.77) | 0.001 |
| Rectal complaints | | 0.12 (0.02 - 0.83) | 0.16 (0.02 - 1.16) | 0.071 |
| Back pain | | 0.32 (0.18 - 0.57) | 0.52 (0.29 - 0.94) | 0.03 |
| Strange of suicidal behaviour | | 2.52 (1.89 - 3.38) | 1.46 (1.06 - 2.01) | 0.022 |
| ABCD unstable | | 7.72 (5.45 - 10.94) | 2.23 (1.49 - 3.32) | <0.001 |
| Arm or leg complaints | | 0.37 (0.26 - 0.53) | 0.67 (0.46 - 0.97) | 0.034 |
| Antibiotics prescribed <72h | | 3.51 (2.77 - 4.45) | 1.29 (0.99 - 1.69) | 0.058 |
