# Supplementary material for: Predicting sepsis-related mortality and ICU admissions from telephone triage information of patients presenting to out-of-hours GP cooperatives with acute infections: A cohort study of linked routine care databases
Source: PLoS One. 2023 Dec 13;18(12):e0294557. doi: 10.1371/journal.pone.0294557 (PMC10718413; doi:10.1371/journal.pone.0294557)
Supplement: S3 Table — (DOCX) [file pone.0294557.s009.docx]

**S9 Table. C-statistic, Brier score and slope of the predicted probabilities of the different models for the composite primary and secondary outcome in the test data (n=50,932).**

| **Model** | **C-statistic** | **Brier score** | **Slope** |
| --- | --- | --- | --- |
| Logistic regression | 0.853 | 0.9852 | 0.8258 |
| Random forest (XGBoost) | 0.849 | 0.9858 | 0.9819 |
| Neural networks | 0.851 | 0.9886 | 1.369 |
