# Supplementary material for: Predicting sepsis-related mortality and ICU admissions from telephone triage information of patients presenting to out-of-hours GP cooperatives with acute infections: A cohort study of linked routine care databases
Source: PLoS One. 2023 Dec 13;18(12):e0294557. doi: 10.1371/journal.pone.0294557 (PMC10718413; doi:10.1371/journal.pone.0294557)
Supplement: S4 Table — (DOCX) [file pone.0294557.s010.docx]

**S10 Table. Diagnostic performance measures at different thresholds of the predicted probabilities of the logistic regression model for the composite primary and secondary outcome in the test data (n=50,932).**

| **Threshold of predicted probability** | **Sensitivity** | **Specificity** | **PPV** | **NPV** | **LR+** | **LR-** |
| --- | --- | --- | --- | --- | --- | --- |
| 0.5% | 80 | 77 | 9.4 | 99.2 | 3.4 | 0.27 |
| 0.59% | 77 | 79 | 9.9 | 99.1 | 3.6 | 0.29 |
| 1% | 70 | 84 | 11 | 98.9 | 4.3 | 0.36 |
| 2% | 57 | 90 | 14 | 98.6 | 5.4 | 0.48 |
| 3% | 46 | 93 | 16 | 98.3 | 6.4 | 0.58 |
| 4% | 35 | 95 | 18 | 98.0 | 7.1 | 0.68 |
| 5% | 27 | 97 | 19 | 97.8 | 7.8 | 0.76 |
| PPV, positive predictive value; NPV, negative predictive value; LR+, positive likelihood ratio; LR-, negative likelihood ratio | | | | | | |
