# Supplementary material for: Predicting sepsis-related mortality and ICU admissions from telephone triage information of patients presenting to out-of-hours GP cooperatives with acute infections: A cohort study of linked routine care databases
Source: PLoS One. 2023 Dec 13;18(12):e0294557. doi: 10.1371/journal.pone.0294557 (PMC10718413; doi:10.1371/journal.pone.0294557)
Supplement: Attachment — Submitted filename: Predicting sepsis_Review.docx [file pone.0294557.s011.docx]

**Predicting sepsis-related mortality and ICU admissions from telephone triage information of patients presenting to out-of-hours GP cooperatives with acute infections: a cohort study of linked routine care databases**

Title is good but the objective or aim is mixed up with the development of model.

Need to explain more about the time frame of data. Why up to 30th Nov, 2019 should have a better explanation.

If it's retrospective cohort study then unexposed group of population at risk was not mentioned clearly. Is it survey or cohort study should be justified.

Discussion is too short related to findings, should be more explanatory with other study findings.

“In conclusion, sepsis-related adverse outcomes can be predicted based on information available during telephone triage but, based on the currently available data, not accurately enough to be of added value in clinical practice.”

Confusing conclusion as the title was not focused in development of any tool related to clinical practice.

Development of an accurate prediction model based on telephone triage information and whether this model can be used to implement a “sepsis alert” was not in title.
